# First report *Xenocorixa vittipennis* (Horváth) (Hemiptera, Corixoidea, Corixidae) from South Korea and checklist of the Korean Corixoidea

**DOI:** 10.3897/BDJ.13.e165218

**Published:** 2025-11-26

**Authors:** Jiseung Kim, Sora Kim

**Affiliations:** 1 Laboratory of Insect Phylogenetics and Evolution, Jeonbuk National University, Jeonju, Republic of Korea Laboratory of Insect Phylogenetics and Evolution, Jeonbuk National University Jeonju Republic of Korea

**Keywords:** aquatic bugs, Heteroptera, new record, taxonomy, water boatmen

## Abstract

**Background:**

*Xenocorixa* Hungerford, 1947 is a monotypic genus containing a single species, *Xenocorixa vittipennis* (Horváth, 1879), known only from China (Beijing, Hubei, Shandong, Shanghai, Tianjin, Zhejiang), Japan (Honshu, Shikoku, Kyushu) and Taiwan.

**New information:**

*Xenocorixa vittipennis* (Horváth, 1879) is reported from South Korea for the first time. A diagnosis and supplementary description of this species are provided, together with illustrations of the adult and genitalia. A revised checklist of Korean Corixoidea is also provided.

## Introduction

Corixoidea Leach, 1815 is one of the largest superfamilies of aquatic bugs (Nepomorpha), comprising approximately 35 genera and more than 600 species (Schuh and Slater 2020). Members of the Corixoidea are found worldwide and are the only water bugs that reach the Arctic (Hungerford 1948, Aukema et al. 1995). The Corixoidea includes three families: the Corixidae Leach, 1815 as proposed by Leach (1815); the Diaprepocoridae Lundblad, 1928, as established by Lundblad (1928) and the Micronectidae Jaczewski, 1924, as delineated by Jaczewski (1924). The classification status of the Diaprepocoridae and Micronectidae has been discussed in numerous studies (see Andersen and Weir (2004), Chen et al. (2005), Schuh and Slater (2020), Ye et al. (2020)). The phylogenetic analyses conducted by Ye et al. (2023) provided consistent support for the monophyly of each family.

*Xenocorixa* Hungeford, 1947 is a monotypic genus in the Corixidae. *Xenocorixa vittipennis* (Horváth, 1879) is the only species in this genus and is distributed in China and Japan (Hayashi and Miyamoto 2018, Xie et al. 2024). This genus is similar to *Hesperocorixa* Kirkaldy, 1908 in general appearance. The two genera can be distinguished by whether or not the lateral lobe of the prothorax is obscured by the mesoepimeron and by differences in the length-to-width ratio of the mesoepimeron.

Studies on the fauna of Corixidae in Korea have been conducted to a very limited extent. The first record of this family from Korea was by Okamoto 1924, who reported *Hesperocorixa distanti* (Kirkaldy, 1899) and Sigara (Tropocorixa) substriata (Uhler, 1896). Kwon et al. (2001) revised Korean Corixidae into three subfamilies (including Micronectinae), four genera and 16 species. After Kwon et al. (2001), there is no taxonomic research about Korean Corixidae and the Micronectidae is still treated as the subfamily Micronectinae in the checklists (Kwon et al. 2001, Cho and Kwon 2017, Park and Lee 2021). The aim of the study is to report *Xenocorixa vittipennis*, which is new to the Korean fauna. Comprehensive description and illustration of the species are provided. A revised checklist of Corixoidea from the Korean Peninsula is also provided herein.

## Materials and methods

Materials for the study were collected for this study using a light trap (mercury vapour lamp, 220V/400W) and a stainless-steel strainer. The specimens were euthanised and preserved in 80% ethanol. Species identification was based on the keys and/or descriptions of Horváth (1879), Hungerford (1947) and Hayashi and Miyamoto (2018). All materials are deposited in the Laboratory of Insect Phylogenetics & Evolution, Jeonbuk National University (JBNU in Jeonju, South Korea) and the National Institute of Biological Resources (NIBR in Incheon, South Korea).

Genital preparations were made with 10% potassiun hydroxide (KOH) at 70℃ for 10 minutes. After rinsing the KOH with distilled water, the genitalia were dissected from the apex of the abdomen and then transferred to glycerine for further examination. The specimens and dissected genitalia were observed using a Canon EOS 6D digital camera with a Canon Macro Lens EF 100 mm (Canon, Japan), mounted on a Stack Shot Macro Package and dome illuminator Lecia LED 5000 HDI or a Tucsen Dhyana 400 DC digital camera (Tucsen Photonics, China) with Leica S8AP0 stereomicroscope (Leica Micoro systems, Germany) with the same dome illuminator. Photographs were taken and merged using Helicon Focus software (version 8.2.2. Pro, Helicon Soft, Ukraine) or Mosaic software (version 2.4) with Adobe Photoshop 2022 (version 23.5.1, Adobe, United States of America). Further detailed images of the antenna and genitalia were captured by utilising a Leica Z16 APO stereomicroscope (Leica Microsystems, Germany) with Optview software (Korea Lab Tech, Republic of Korea). The distribution map was created using QGIS 3.40.4 (OGSeo, Switzerland). For the information on the distribution map, we cited Xie et al. (2024) for China and the Red Data Books of each Prefecture and Hasegawa (2018) for Japan.

## Taxon treatments

### Xenocorixa

Hungeford, 1947

9047E681-D3B1-55F7-A14C-EB4D5903E515

Xenocorixa Hungeford, 1947 - Hungerford (1947): 93.
Corisa vittipennis Horváth, 1879

### Xenocorixa vittipennis

Horváth, 1879

25ED0F02-BC79-5FD8-8563-55A468F4AB8E

Corisa vittipennis Horváth, 1879 - Horváth (1879): 151. Type locality: China, Sobsam-Timbu.
Corisa miyakei Matsumura, 1905 - Matsumura (1905): 64. Type locality: Japan, Nakano nr Tokyo.
Sigara horni Jaczewski, 1928 - Jaczewski (1928): 110. Type locality: China, Shantun, Yenchowfu.
Sigara vittipennis : Lundblad, 1933a - Lundblad (1933): 451.
Xenocorixa vittipennis : Hungerford, 1947 - Hungerford (1947): 93.

#### Materials

- **Type status:** Other material. **Occurrence:** recordedBy: J.-W. Cheong; individualCount: 1; sex: female; lifeStage: adult; occurrenceID: D6551C7A-6003-5ECE-B013-EFB347211D43; **Taxon:** kingdom: Animalia; phylum: Arthropoda; class: Insecta; order: Hemiptera; family: Corixidae; genus: Xenocorixa; specificEpithet: vittipennis; **Location:** country: South Korea; stateProvince: Gyeongsangnam-do; county: Hadong-gun; locality: Geumnam-myeon, Daesong-ri, Daesongsoryuji; verbatimElevation: 146 m; decimalLatitude: 34.977784; decimalLongitude: 127.858864; **Event:** eventDate: 7 VII 2010; **Record Level:** institutionCode: NIBR
- **Type status:** Other material. **Occurrence:** recordedBy: J. Kim et al.; individualCount: 21; sex: 8 males, 13 females; lifeStage: adult; occurrenceID: 419DA9EA-197A-58D7-9BA6-9B2EDA122EAE; **Taxon:** kingdom: Animalia; phylum: Arthropoda; class: Insecta; order: Hemiptera; family: Corixidae; genus: Xenocorixa; specificEpithet: vittipennis; **Location:** country: South Korea; stateProvince: Jeollanam-do; county: Wando-gun; locality: Wando-eup, Jeongdo-ri; verbatimElevation: 8 m; decimalLatitude: 34.309312; decimalLongitude: 126.705769; **Event:** eventDate: 26 VII 2024; **Record Level:** institutionCode: JBNU
- **Type status:** Other material. **Occurrence:** recordedBy: J. Kim and D. Choi; individualCount: 1; sex: male; lifeStage: adult; occurrenceID: A563CB13-359F-5EA2-AE7D-17652FF0D41E; **Taxon:** kingdom: Animalia; phylum: Arthropoda; class: Insecta; order: Hemiptera; family: Corixidae; genus: Xenocorixa; specificEpithet: vittipennis; **Location:** country: South Korea; stateProvince: Incheon; county: Ongjin-gun; locality: Deokjeok-myeon, Seopo-ri; verbatimElevation: 52 m; decimalLatitude: 37.214759; decimalLongitude: 126.116845; **Event:** eventDate: 13 VIII 2024; **Record Level:** institutionCode: JBNU
- **Type status:** Other material. **Occurrence:** recordedBy: J. Kim; individualCount: 45; lifeStage: 2 2nd instar nymphs, 43 3rd instar nymphs; occurrenceID: FAA01C47-554F-5CEB-9708-1EFE59C2979F; **Taxon:** kingdom: Animalia; phylum: Arthropoda; class: Insecta; order: Hemiptera; family: Corixidae; genus: Xenocorixa; specificEpithet: vittipennis; **Location:** country: South Korea; stateProvince: Jeollanam-do; county: Wando-gun; locality: Wando-eup, Jeongdo-ri; verbatimElevation: 8 m; decimalLatitude: 34.309312; decimalLongitude: 126.705769; **Event:** eventDate: 29 II 2025; **Record Level:** institutionCode: JBNU
- **Type status:** Other material. **Occurrence:** recordedBy: J. Kim et al.; individualCount: 1; sex: male; lifeStage: adult; occurrenceID: 32F21793-3B14-5517-B0D3-77DE2209940F; **Taxon:** kingdom: Animalia; phylum: Arthropoda; class: Insecta; order: Hemiptera; family: Corixidae; genus: Xenocorixa; specificEpithet: vittipennis; **Location:** country: South Korea; stateProvince: Jeollanam-do; county: Hampyeong-gun; locality: Hampyeong-eup, Japung-ri; verbatimElevation: 113 m; decimalLatitude: 35.036441; decimalLongitude: 126.468439; **Event:** eventDate: 23 VI 2025; **Record Level:** institutionCode: JBNU

#### Description

**Body length.** ♂ 8.3–8.8 mm; ♀ 8.7–9.3 mm (based on 24 specimens).

**Figure 1.**
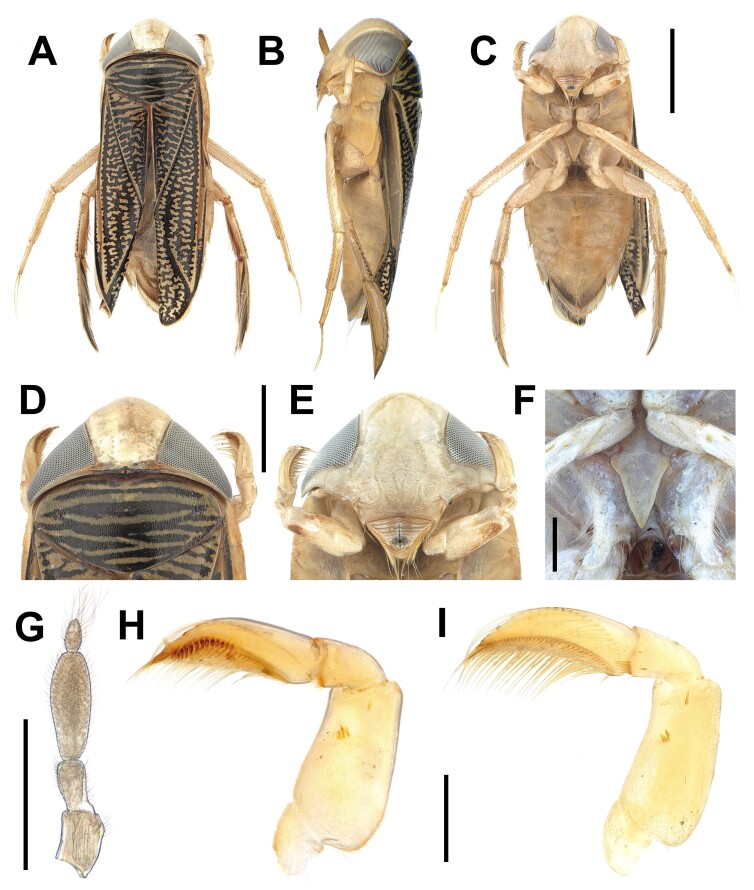
*Xenocorixa vittipennis* (Horváth, 1879). **A–C** habitus of male in (A) dorsal, (B) lateral, (C) ventral; **D–E** head and pronotal disk of male in (D) dorsal, (E) ventral; F, metaxyphus; **G** antenna; **H** foreleg of male; **I** foreleg of female. Scale bars: A–C, 2 mm; D–E, 1 mm; F–I, 0.5 mm.

**Head** (Fig. 1D, E and G). Whitish-yellow. Head slightly longer than the pronotal disc in dorsal view, its anterior margin protruding beyond the eyes. Male fovea depressed. Infraocular area reduced. Rostrum with transverse sulcations. The antennae four segments I–IV; antennal segments I–II shorter; antennal segment IV one-third the length of the antennal segment III.

**Thorax** (Fig. 1D–F, H and I). Yellowish-brown. Pronotum with distinct 6–7 dark transverse bands. Mesoepimeron raised broadly. Hemelytra with reticulation of dark lines; membrane narrow. Fore-leg similar in male and female; front tibia short; pala long, flat, densely covered with palmar hairs in ventral side; male with peg row consisting of 14 pegs. Mid-leg with 2 apical claws; claws longer than tarsus. Hind-leg shorter than mid-leg; tarsus densely covered with swimming hairs. Metaxyphus triangular.

**Figure 2.**
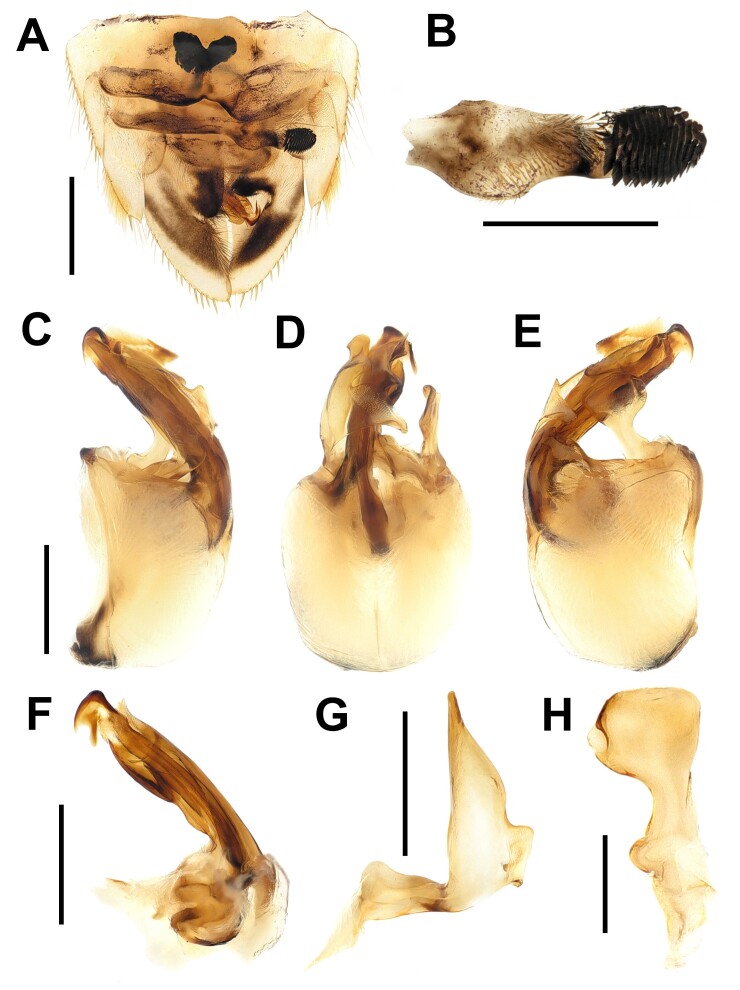
Detail abdominal and genitalic features of *Xenocorixa vittipennis*. **A** tergite V–VIII of male in dorsal; **B** strigil; **C–E** genital capsule of male in (C) dorsal, (D) lateral, (E) ventral; **F** aedeagus in dorsal; **G** left paramere in dorsal; **H** right paramere in dorsal. Scale bars: A, 1 mm; B–G, 0.5 mm; H, 0.25 mm.

**Abdomen** (Fig. 2). Yellowish-brown. Male abdominal asymmetry dextral. Strigil consisting of 14 combs. Genital capsule yellowish-brown, oval in lateral view; aedeagus apical furcate; right paramere strongly broader median to apex; left paramere strongly curved at basal 1/3, tapering apically.

#### Diagnosis

Lateral lobe of prothorax is obscured posteriorly by mesoepimeron. Mesoepimeron is shorter than width in lateral view, with a secondary suture extending across it from the lateral bend to the inner basal angle of the mesoepimeron. The last antennal segment very short, one-third the length of the antennal segment III. The pruinose area along the base of the claval suture is short and broad. Males with dextral strigil (Hungerford 1947, Hasegawa 2018).

#### Distribution

China (North: Beijing, Tianjin; East: Shandong, Shanghai, Zhejiang; Central: Hubei (Xie et al. 2024)), Japan (Honshu, Shikoku, Kyushu (Nakajima et al. 2020)), Korea (new record), Taiwan (Fig. 4).

#### Notes

**Figure 3.**
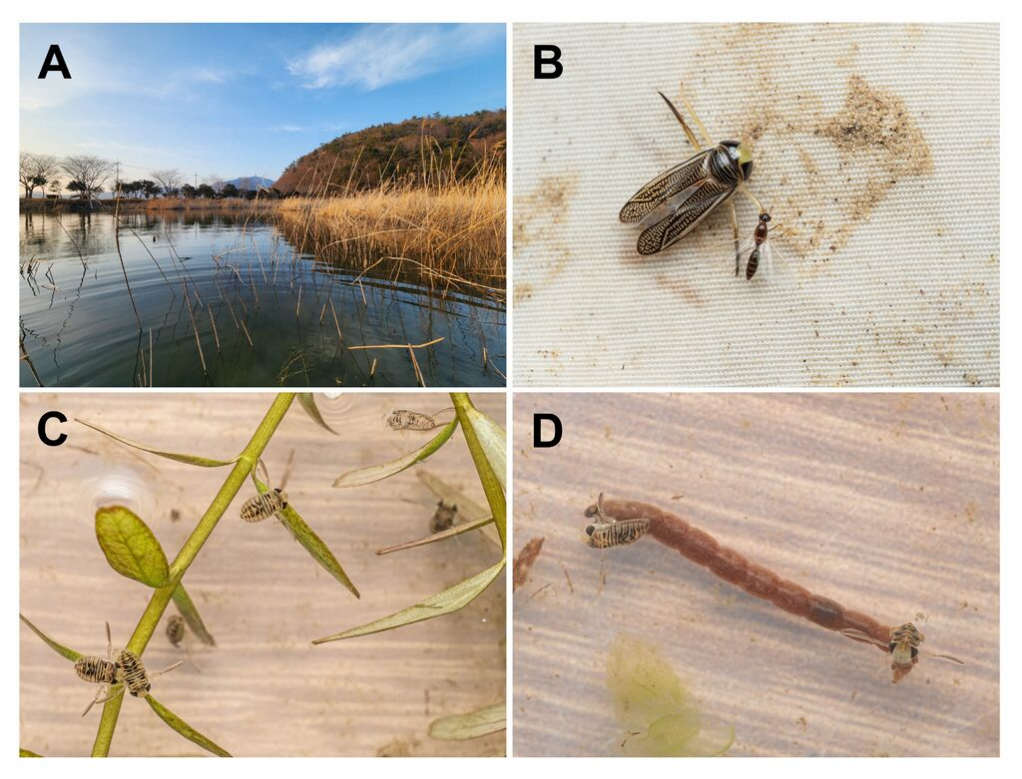
**A** habitat (Wando-gun, South Jeolla Province); **B** female adult attracted to light trap; **C** 3^rd^ instar nymphs; **D** 3^rd^ instar nymphs feeding on frozen bloodworm.

**Figure 4.**
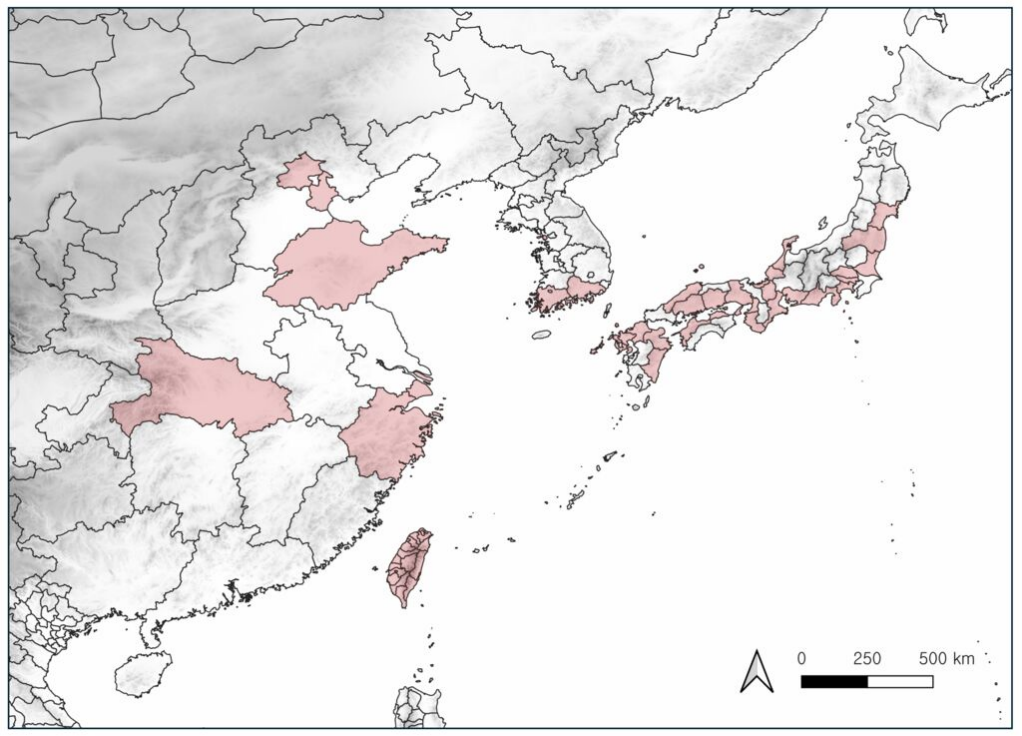
Distribution of *Xenocorixa vittipennis* in east Asia.

**Figure 5.**
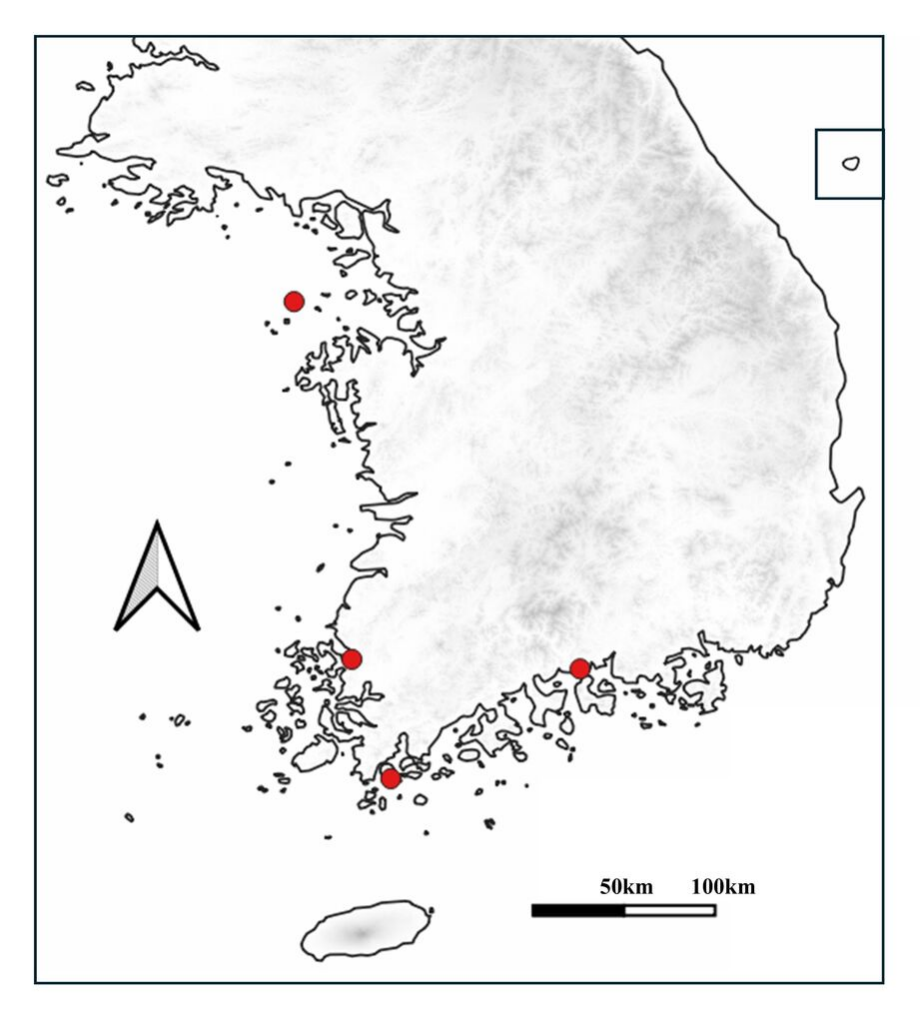
Detailed distribution of *Xenocorixa vittipennis* in South Korea.

*Xenocorixa vittipennis* was observed in an agricultural reservoir (Fig. 3A) from South Korea near the coastal area (Fig. 5). Adults demonstrated a positive phototactic response and were collected through light traps (Fig. 3B). In February, 2^nd^ and 3^rd^ instar nymphs were collected in an agricultural reservoir in Wando-gun (Fig. 3C). Meanwhile, reports from Miyagi Prefecture, Japan, suggest that the species overwinters as eggs that hatch the following spring (Hasegawa 2018).

Most of the aquatic bugs are carnivorous. On the other hand, while zoophagy is the dominant feeding strategy in Corixidae, many species also consume other foods such as algae and detritus and their diet can vary significantly amongst different species, sexes or even populations of a single species, based on food availability (Haedicke et al. 2017, Xie et al. 2024). In this study, we documented the feeding behaviour of the 2^nd^ and 3^rd^ instar larvae of *Xenovorixa vittipennis* on frozen bloodworms under laboratory conditions (Fig. 3D).

## Checklists

### A checklist of Korean Corixoidea

#### Corixoidea

Leach, 1815

305774D5-CD1D-5FE2-83D6-1CF34C1C0314

#### Corixidae

Leach, 1815

06070038-0C0B-5FFE-A3E1-F046D92A110B

#### Corixinae

Leach, 1815

2D204EE1-60F3-5A23-B374-C13EC19EE7FD

#### Hesperocorixa

Kirkaldy, 1908

6EBBDFF8-7D4B-5BEE-9FC7-1CD8FF5F9EA9

#### Hesperocorixa crassipala

(Hungerford, 1940)

E9C3A4E3-EA9D-56B0-90BC-485E416157A3

##### Distribution

Korea, China (Henan, Hubei, Jiangsu, Shaanxi), Vietnam (Xie et al. 2024).

#### Hesperocorixa distanti

(Kirkaldy, 1899)

12780910-7E0D-5840-8131-C802CD599D33

##### Distribution

Korea (Gyeonggi-do, Chungcheongnam-do, Gyeongsangbuk-do, Gyeonsangnam-do, Jeju-do), China (Guangxi Zhuang Autonomous Region, Guizhou, Hebei, Henan, Hubei, Jiangsu, Jiangxi, Shanghai), Japan (Hokkaido, Honshu), Russia (Far East) (Okamoto 1924, Doi 1935, Lee and Kwon 1991, Kwon et al. 2001, Hayashi and Miyamoto 2018, Xie et al. 2024)

#### Hesperocorixa hokkensis

(Matsumura, 1905)

2D7FDAA0-5F8F-570B-B2BD-99319526D448

##### Distribution

Korea (Gyeonggi-do, Gyeongsangbuk-do, Gyeongsangnam-do), Japan (Honshu, Shikoku, Kyushu) (Lee 1971, Nam 1973, Kwon et al. 2001, Cho and Kwon 2017, Hayashi and Miyamoto 2018).

#### Hesperocorixa mandshurica

(Jaczewski, 1924)

6EACA771-F89E-5EC2-8A58-D07FED05DA64

##### Materials

- **Type status:** Other material. **Occurrence:** recordedBy: J. Park; individualCount: 4; sex: 2 males, 2 females; lifeStage: adult; occurrenceID: 91815FFE-0E3F-5592-BB03-C97D15A62D49; **Taxon:** genus: Hesperocorixa; specificEpithet: mandshurica; **Location:** country: South Korea; stateProvince: Gyeongsangnam-do; county: Changnyeong-gun; locality: Yueo-myeon, Sejin-ri, Upo Wetland; verbatimElevation: 85 m; decimalLatitude: 35.544722; decimalLongitude: 128.422500; **Event:** eventDate: 1 VI 2009; **Record Level:** institutionCode: NIBR
- **Type status:** Other material. **Occurrence:** recordedBy: H. Kim; individualCount: 2; sex: 2 males; lifeStage: adult; occurrenceID: 1BE7858D-7E64-54E2-AE71-D88D74EA8747; **Taxon:** genus: Hesperocorixa; specificEpithet: mandshurica; **Location:** country: South Korea; stateProvince: Gangwon-do; county: Hongcheon-gun; locality: Duchon-myeon, Cheonhyeon-ri, Mt. Gari; **Event:** eventDate: 30 VI 2016; **Record Level:** institutionCode: NIBR
- **Type status:** Other material. **Occurrence:** recordedBy: J. Kim; individualCount: 1; sex: 1 female; lifeStage: adult; occurrenceID: B2FA7413-6ECB-5726-9C64-2ECF4CBF4705; **Taxon:** genus: Hesperocorixa; specificEpithet: mandshurica; **Location:** country: South Korea; stateProvince: Chungcheongbuk-do; county: Jecheon-si; locality: Cheongpung-myeon, Dan-ri; verbatimElevation: 200 m; decimalLatitude: 36.972222; decimalLongitude: 128.163333; **Event:** eventDate: 13 VI 2024; **Record Level:** institutionCode: JBNU

##### Distribution

Korea (Gangwon-do, Chungcheongbuk-do, Gyeonsangnam-do), China (Beijing, Hebei, Heilongjiang, Henan, Inner Mongolia Autonomous Region, Jiangsu, Jilin, Liaoning, Shaanxi, Shandong, Shanxi, Tianjin), Japan (Honshu, Shikoku, Kyushu), Russia (Far East) (Hayashi and Miyamoto 2018, Xie et al. 2024).

#### Corixini

Leach, 1815

A35858C3-4811-5B21-8CAD-CD8A2089DFDD

#### Sigara

Fabricius, 1775

547293FD-5CC8-5C19-B71F-01E762EE0BED

#### Pseudovermicorixa

Jaczewski, 1962

616D0BB1-43AD-5B6D-9C09-9B1758A68850

#### Sigara (Pseudovermicorixa) septemlineata

(Paiva, 1918)

95FE5D0F-12E1-5436-BF3B-8C4E1097C9F2

##### Materials

- **Type status:** Other material. **Occurrence:** recordedBy: J. Kim; individualCount: 6; sex: 1 males, 5 females; lifeStage: adult; occurrenceID: 726EE717-8AFB-5843-A869-F73E7B89D913; **Taxon:** genus: Sigara; subgenus: Pseudovermicorixa; specificEpithet: septemlineata; **Location:** country: South Korea; stateProvince: Jeollanam-do; county: Jangseong-gun; locality: Seosam-myeon, Songhyeon-ri; verbatimElevation: 79 m; decimalLatitude: 35.357500; decimalLongitude: 126.770556; **Event:** eventDate: 29 IX 2023; **Record Level:** institutionCode: JBNU
- **Type status:** Other material. **Occurrence:** recordedBy: J. Kim; individualCount: 2; sex: 1 males, 1 females; lifeStage: adult; occurrenceID: 0FE6841B-7AE9-5307-B371-C9A11D958D99; **Taxon:** genus: Sigara; subgenus: Pseudovermicorixa; specificEpithet: septemlineata; **Location:** country: South Korea; stateProvince: Jeollanam-do; county: Wando-gun; locality: Sinji-myeon, Sin-ri; verbatimElevation: 6 m; decimalLatitude: 34.328333; decimalLongitude: 126.822222; **Event:** eventDate: 1 VII 2025; **Record Level:** institutionCode: JBNU

##### Distribution

Korea (South: Gangwon-do, Gyeongsangbuk-do, Gyeongsangnam-do, Jellanam-do; North: Pyongyang), China (Fujian, Guizhou, Heilongjiang, Hongkong, Hubei, Inner Mongolia Autonomous Region, Guangxi Zhuang Autonomous Region, Jiangxi, Shaanxi, Shandong, Shanxi, Sichuan, Yunnan), India, Japan (Honshu, Shikoku, Kyushu, Nansei Islands Tsushima Island), Russia (Far East), Myanmar, Taiwan (Jaczewski 1963, Kwon et al. 2001, Hayashi and Miyamoto 2018, Xie et al. 2024).

#### Sigara

Fabricius, 1775

4E342AA0-E35F-5935-B006-8754BFEB9B0A

#### Sigara (Sigara) weymarni

Hungerford, 1940

8E93721F-38B1-5DB2-B033-1019D45379B9

##### Distribution

Korea, China (Heilongjiang, Inner Mongolia Autonomous Region, Jilin, Liaoning), Mongolia, Russia (Xie et al. 2024).

#### Tropocorixa

Hutchinson, 1940

CB77AEEC-5FDD-53B3-BBAC-3E6B2C34B44E

#### Sigara (Tropocorixa) bellula

(Horváth, 1879)

F0FF9FD3-CD4F-54BE-A079-8DA52D3BFD1F

##### Materials

- **Type status:** Other material. **Occurrence:** recordedBy: J. Kim; individualCount: 13; sex: 2 males, 11 females; lifeStage: adult; occurrenceID: 68FF6ACA-6F77-570F-9F23-4666D418E09A; **Taxon:** genus: Sigara; subgenus: Tropocorixa; specificEpithet: septemlineata; **Location:** country: South Korea; stateProvince: Gyeonggi-do; county: Hwaseong-si; locality: Songsan-myeon, Gojeong-ri; verbatimElevation: 2 m; decimalLatitude: 37.256111; decimalLongitude: 126.745833; **Event:** eventDate: 1 VII 2024; **Record Level:** institutionCode: JBNU
- **Type status:** Other material. **Occurrence:** recordedBy: J. Kim; individualCount: 2; sex: 1 males, 1 females; lifeStage: adult; occurrenceID: 28EB4C4B-F7E8-5A91-ADE8-9C05A96B6C15; **Taxon:** genus: Sigara; subgenus: Tropocorixa; specificEpithet: bellula; **Location:** country: South Korea; stateProvince: Jellanam-do; county: Wando-gun; locality: Sinji-myeon, Sin-ri; verbatimElevation: 6 m; decimalLatitude: 34.328333; decimalLongitude: 126.822222; **Event:** eventDate: 1 VII 2025; **Record Level:** institutionCode: JBNU

##### Distribution

Korea (Gyeonggi-do, Jellanam-do), China (Anhui, Guangxi Zhuang Autonomous Region, Guizhou, Henan, Hubei, Hunan, Inner Mongolia Autonomous Region, Jiangsu, Jiangxi, Ningxia Hui Autonomous Region, Shaanxi, Shanxi, Tianjin, Zhejiang), Japan (Honshu, Kyushu, Tsushima Island), Russia, Taiwan (Hayashi and Miyamoto 2018, Xie et al. 2024).

#### Sigara (Tropocorixa) formosana

(Matsumura, 1915)

37A79432-EE8B-5A01-8E47-1462C9181CB9

##### Distribution

Korea (Gyeonggi-do), Japan (Nasei Islands), Taiwan, Vietnam (Lee 1971, Hayashi and Miyamoto 2018, Xie et al. 2024).

#### Sigara (Tropocorixa) gaginae

Jaczewski, 1960

86EA7F8C-630B-585E-B3F0-00C751E3AA17

##### Distribution

Korea, China (Anhui, Guizhou Heilongjiang, Hubei, Hunan, Inner Mongolia Autonomous Region, Jiangsu, Jiangxi, Jilin, Liaoning, Zhejiang), Russia (Xie et al. 2024).

#### Sigara (Tropocorixa) nigroventralis

(Matsumura, 1905)

ECE76DBB-CBF7-5871-9F3D-4C00307092C7

##### Distribution

Korea (Pyongyang), Japan (Hokkaido, Honshu, Shikoku, Kyushu, Tsushima Island, Nansei Islands), Russia (Far East), Taiwan (Jaczewski 1963, Cho and Kwon 2017, Hayashi and Miyamoto 2018).

#### Sigara (Tropocorixa) paivai

Lundblad, 1928

6AE973C6-CE1F-5E52-AA8C-62D4CC2A9B14

##### Distribution

Korea, Sumatra, Thailand, India (Kwon et al. 2001).

#### Sigara (Tropocorixa) substriata

(Uhler, 1896)

C9A55817-E254-590A-80B3-409F542EA6C8

##### Distribution

Korea (South: Gangwon-do, Gyeonggi-do, Gyeongsangbuk-do, Gyeongsangnam-do, Jeollabuk-do, Jellanam-do, Jeju-do; North: Pyongyang), China (Heilongjiang, Henan, Zhejiang), Japan (Hokkaido, Honshu, Shikoku, Kyushu), Russia (Far East) (Kwon et al. 2001, Hayashi and Miyamoto 2018, Xie et al. 2024).

#### Xenocorixa

Hungerford, 1947

DECBCD87-AD87-537A-A32C-6C80B9194D0B

#### Xenocorixa vittipennis

(Horváth, 1879)

9C96A569-74BF-5D57-9677-69A0D36F35E7

##### Distribution

Korea (Incheon, Gyeongsangnam-do, Jellanam-do), China (Beijing, Hubei, Shandong, Shanghai, Tianjin, Zhejiang), Japan (Honshu, Shikoku, Kyushu), Taiwan (Hayashi and Miyamoto 2018, Xie et al. 2024).

#### Cymatiainae

Walton, 1940

31CEEC07-EF1F-58ED-951C-3C133F20C8F4

#### Cymatia

Flor, 1860

F4C8D778-3461-5094-9A34-EB460919A5ED

#### Cymatia apparens

(Distant, 1911)

37112751-8647-5589-92A4-5F91299B0DA1

##### Materials

- **Type status:** Other material. **Occurrence:** recordedBy: C. Lee; individualCount: 1; sex: male; lifeStage: adult; occurrenceID: 7F70745C-88C8-5C6A-B2C1-D92D9CF6F61A; **Taxon:** genus: Cymatia; specificEpithet: apparens; **Location:** country: South Korea; stateProvince: Gyeongsangnam-do; county: Tongyeong-si; locality: Gwangdo-myeon, Nosan-ri; **Event:** eventDate: 31 IV 1964; **Record Level:** institutionCode: NIBR
- **Type status:** Other material. **Occurrence:** recordedBy: J. Kim; individualCount: 23; sex: 11 males, 12 females; lifeStage: adult; occurrenceID: EBD3FA00-632E-5A8F-95D7-493D92EE9A13; **Taxon:** genus: Cymatia; specificEpithet: apparens; **Location:** country: South Korea; stateProvince: Gyeongsangbuk-do; county: Ulleung-gun; locality: Buk-myeon, Hyeonpo-ri; verbatimElevation: 192m; decimalLatitude: 37.517156; decimalLongitude: 130.815867; **Event:** eventDate: 12 VII 2024; **Record Level:** institutionCode: NIBR

##### Distribution

Korea (Gyeongsangbuk-do, Gyeongsangnam-do), China (Beijing, Guizhou, Henan, Hubei, Hunan, Inner Mongolia Autonomous Region, Jiangsu, Jiangxi, Shaanxi, Shandong, Shanxi, Tianjin, Zhejiang), India, Japan (Hokkaido, Honshu, Shikoku, Kyushu), Russia (Far East) (Hayashi and Miyamoto 2018, Xie et al. 2024).

#### Micronectidae

Jaczewski, 1924

E5B36852-C1E4-5A7A-A694-DF81D041B083

#### Micronectinae

Jaczewski, 1924

1FC39D4E-3574-58CA-9990-0F149530BC4E

#### Micronecta

Kirkaldy, 1897

3B6FFAFC-1D1B-5C6D-9BDA-A2AAAF0C8319

#### Basileonecta

Hutchinson, 1940

952F68AE-53A9-5165-9E7C-513543605AB3

#### Micronecta (Basileonecta) sahlbergii

(Jakovlev, 1881)

397FCC54-EF59-5426-80C3-BD76160A8C9A

##### Materials

- **Type status:** Other material. **Occurrence:** recordedBy: J. Kim; individualCount: 15; sex: 3 males, 12 females; lifeStage: adult; occurrenceID: A6E485A0-D690-5EE6-9EB6-78C8BFC8F2EF; **Taxon:** genus: Micronecta; subgenus: Basileonecta; specificEpithet: sahlbergii; **Location:** country: South Korea; stateProvince: Jeollanam-do; county: Gochang-gun; locality: Buan-myeon, Sudong-ri; verbatimElevation: 84 m; decimalLatitude: 35.537048; decimalLongitude: 126.651914; **Event:** eventDate: 7 VII 2025; **Record Level:** institutionCode: JBNU

##### Distribution

Korea (South: Jeollanam-do; North: Pyongyang), China (Anhui, Guangdong, Guizhou, Hainan, Hebei, Heilongjiang, Henan, Hubei, Hunan, Inner Mongolia Autonomous Region, Jiangsu, Jiangxi, Shandong, Shannxi, Shanxi, Sichuan, Tianjin, Yunnan, Zhejiang), Iran, Japan (Honshu, Shikoku, Kyushu, Nasei Islands), Russia (Far East), Taiwan, Vietnam (Jaczewski 1963, Hayashi and Miyamoto 2018, Xie et al. 2024).

#### Micronecta (Basileonecta) sedula

Horváth, 1905

8D7FDC2E-6463-504D-A147-EBF20F7EE0FF

##### Distribution

Korea (South: Chungcheongnam-do, Gyeongsangbuk-do, Jellabuk-do, Jeju-do, North: Pyongyang), China (Anhui, Hubei, Hunan, Inner Mongolia Autonomous Region, Jiangsu, Jiangxi, Tianjin, Zhejiang), Japan (Honshu, Shikoku, Kyushu), Russia (Far East), Vietnam (Jaczewski 1963, Lee and Kwon 1991, Kim 1993, An 1998, Hayashi and Miyamoto 2018, Xie et al. 2024).

#### Micronecta

Kirkaldy, 1897

E6F0A46E-7FCE-52FE-AA45-4C6CA72C8941

#### Micronecta (Micronecta) guttata

Matsumura, 1905

FB4CD120-D26F-56B2-B97F-16A8F031A722

##### Materials

- **Type status:** Other material. **Occurrence:** recordedBy: J. Kim; individualCount: 2; sex: 2 females; lifeStage: adult; occurrenceID: 7F76F09A-9C19-5120-BB15-23CB7E3DBCE8; **Taxon:** genus: Micronecta; subgenus: Micronecta; specificEpithet: guttata; **Location:** country: South Korea; stateProvince: Gwangju; county: Buk-gu; locality: Sinyong-dong; verbatimElevation: 19 m; decimalLatitude: 35.217778; decimalLongitude: 126.862222; **Record Level:** institutionCode: JBNU

##### Distribution

Korea (Gyeongsangbuk-do, Gwangju), China (Fujian, Guangdong, Guangxi Zhuang Autonomous Region, Inner Mongolia Autonomous Region), Japan (Honshu, Shikoku, Kyushu), Kazakhstan, Mongolia, Russia (Far East, East Siberia), Taiwan (Lee and Kwon 1991, Cho and Kwon 2017, Hayashi and Miyamoto 2018, Xie et al. 2024).

## Supplementary Material

XML Treatment for Xenocorixa

XML Treatment for Xenocorixa vittipennis

XML Treatment for Corixoidea

XML Treatment for Corixidae

XML Treatment for Corixinae

XML Treatment for Hesperocorixa

XML Treatment for Hesperocorixa crassipala

XML Treatment for Hesperocorixa distanti

XML Treatment for Hesperocorixa hokkensis

XML Treatment for Hesperocorixa mandshurica

XML Treatment for Corixini

XML Treatment for Sigara

XML Treatment for Pseudovermicorixa

XML Treatment for Sigara (Pseudovermicorixa) septemlineata

XML Treatment for Sigara

XML Treatment for Sigara (Sigara) weymarni

XML Treatment for Tropocorixa

XML Treatment for Sigara (Tropocorixa) bellula

XML Treatment for Sigara (Tropocorixa) formosana

XML Treatment for Sigara (Tropocorixa) gaginae

XML Treatment for Sigara (Tropocorixa) nigroventralis

XML Treatment for Sigara (Tropocorixa) paivai

XML Treatment for Sigara (Tropocorixa) substriata

XML Treatment for Xenocorixa

XML Treatment for Xenocorixa vittipennis

XML Treatment for Cymatiainae

XML Treatment for Cymatia

XML Treatment for Cymatia apparens

XML Treatment for Micronectidae

XML Treatment for Micronectinae

XML Treatment for Micronecta

XML Treatment for Basileonecta

XML Treatment for Micronecta (Basileonecta) sahlbergii

XML Treatment for Micronecta (Basileonecta) sedula

XML Treatment for Micronecta

XML Treatment for Micronecta (Micronecta) guttata
